# Supplementary material for: Encoding of cerebellar dentate neuron activity during visual attention in rhesus macaques
Source: eLife. 2025 Jan 16;13:RP99696. doi: 10.7554/eLife.99696 (PMC11737872; doi:10.7554/eLife.99696)
Supplement: Supplementary file 1. — Only two of the regression models do significantly better than a constant model (F-test, highlighted in bold). Both of those do not survive a Bonferroni correction (α = 0.0083 for 0.05/6 test per monkey). [file elife-99696-supp1.docx]

**Supplementary Material**

**Encoding of cerebellar dentate neuron activity during visual attention in rhesus macaques**

Nico A. Flierman^1,2#^, Sue Ann Koay^3#^, Willem S. van Hoogstraten^2^, Tom J.H. Ruigrok^2^, Pieter R. Roelfsema^1,4,5^, Aleksandra Badura^2*^ and Chris I. De Zeeuw^1,2*^

^1^ Netherlands Institute for Neuroscience, Amsterdam, 1105 BA, The Netherlands

^2^ Department of Neuroscience, Erasmus MC, Rotterdam, 3015 CN, The Netherlands

^3^ Janelia Research Campus, Howard Hughes Medical Institute, Ashburn, VA 20174, USA

^4^ Department of Integrative Neurophysiology, VU University, Amsterdam, 1081 HV, The Netherlands

^5^ Department of Psychiatry, Academic Medical Centre, Amsterdam, 1105 AZ, The Netherlands

^#^ These authors contributed equally to this work.

^*^**Correspondence**:

Aleksandra Badura ([a.badura@erasmusmc.nl](mailto:a.badura@erasmusmc.nl))

Department of Neuroscience, Erasmus MC

Wytemaweg 80, 3015 CN Rotterdam

tel: 0031-(0)10 7043589

Chris I De Zeeuw ([c.de.zeeuw@nin.knaw.nl](mailto:c.de.zeeuw@nin.knaw.nl))

Netherlands Institute for Neuroscience

Meibergdreef 47,1105 BA, Amsterdam

tel: 0031-(0)20- 5665500

| **Mo** | | | | |
| --- | --- | --- | --- | --- |
|  | | Gap L/R n-1 | Gap U/D n-1 | Sac n- 1 |
| LR | R | 0.27 | 0.03 | 0 |
|  | p | **0.03** | 0.47 | 0.98 |
| UD | R | 0 | 0.07 | 0 |
|  | p | 0.93 | 0.3 | 0.97 |
| **Mi** | | | | |
|  | | Gap L/R n-1 | Gap U/D n-1 | Sac n- 1 |
| LR | R | 0 | 0.05 | 0.32 |
|  | p | 0.86 | 0.37 | **0.016** |
| UD | R | 0.02 | 0.11 | 0.13 |
|  | p | 0.59 | 0.19 | 0.16 |

***Supplementary File 1.*** *Regression analyses applied to the data from Supplementary Figure1. Only two of the regression models do significantly better than a constant model (F-test, highlighted in bold). Both of those do not survive a Bonferroni correction (α = 0.0083 for 0.05/6 test per monkey).*
