# Supplementary material for: Encoding of cerebellar dentate neuron activity during visual attention in rhesus macaques
Source: eLife. 2025 Jan 16;13:RP99696. doi: 10.7554/eLife.99696 (PMC11737872; doi:10.7554/eLife.99696)
Supplement: Supplementary file 2. [file elife-99696-supp2.docx]

**Supplementary Material**

**Encoding of cerebellar dentate neuron activity during visual attention in rhesus macaques**

Nico A. Flierman^1,2#^, Sue Ann Koay^3#^, Willem S. van Hoogstraten^2^, Tom J.H. Ruigrok^2^, Pieter R. Roelfsema^1,4,5^, Aleksandra Badura^2*^ and Chris I. De Zeeuw^1,2*^

^1^ Netherlands Institute for Neuroscience, Amsterdam, 1105 BA, The Netherlands

^2^ Department of Neuroscience, Erasmus MC, Rotterdam, 3015 CN, The Netherlands

^3^ Janelia Research Campus, Howard Hughes Medical Institute, Ashburn, VA 20174, USA

^4^ Department of Integrative Neurophysiology, VU University, Amsterdam, 1081 HV, The Netherlands

^5^ Department of Psychiatry, Academic Medical Centre, Amsterdam, 1105 AZ, The Netherlands

^#^ These authors contributed equally to this work.

^*^**Correspondence**:

Aleksandra Badura ([a.badura@erasmusmc.nl](mailto:a.badura@erasmusmc.nl))

Department of Neuroscience, Erasmus MC

Wytemaweg 80, 3015 CN Rotterdam

tel: 0031-(0)10 7043589

Chris I De Zeeuw ([c.de.zeeuw@nin.knaw.nl](mailto:c.de.zeeuw@nin.knaw.nl))

Netherlands Institute for Neuroscience

Meibergdreef 47,1105 BA, Amsterdam

tel: 0031-(0)20- 5665500

| Ramping latency (ms) | Mean  (correct) | St. Dev  (correct) | Mean  (incorrect) | St. Dev  (incorrect) | N  (neurons) |
| --- | --- | --- | --- | --- | --- |
| Mo | 312.8 | 234.1 | 319.8 | 227.0 | 64 |
| Mi | 412.4 | 238.9 | 451.8 | 233.2 | 68 |

***Supplementary File 2.*** *Descriptive statistics of latency to ramping onset.*
